# Use of the results of the study of oral fluid and buccal epithellium in the diagnosis of Alzheimer`s disease

**DOI:** 10.1192/j.eurpsy.2022.1556

**Published:** 2022-09-01

**Authors:** A. Sidenkova

**Affiliations:** Ural State Medical University, Psychiatry, Yekaterinburg, Russian Federation

**Keywords:** ORAL FLUID, BUCCAL EPITHELIUM, Alzheimer’s disease

## Abstract

**Introduction:**

The aim of the study was a comparative analysis of the results of neuropsychological tests with the indicators of the study of oral fluid and buccal epithelium cytograms in patients with Alzheimer’s disease.

**Objectives:**

In the main group of 12 patients with Alzheimer’s disease, m=76.25±4.89. There were 12 cognitively healthy people in the control group. The average MMSE score among the observations of the main group was 13.42±3.63.

**Methods:**

The ADAS-COG scale was used to detail the impaired cognitive functions. The concomitant pathology is compensated. The content of BDNF, TNF-α, IL1RA, IL-6, and IL-8 was determined in the oral fluid and in the blood serum.

**Results:**

When analyzing buccal cytograms, attention was drawn to a pronounced increase in the number of cells with micronuclei in patients with AD to 1.8%; in the control group, the median was 0.1% (p<0.05). A direct correlation was established between the number of binuclear cells and the level of BDNF in the blood serum (r=0.646; p=0.03) in patients with AD. It is also important to note that the level of serum BDNF had a significant direct correlation with immediate memory, and the concentration of salivary BDNF correlates with the parameter of naming objects.

**Conclusions:**

Correlations between amnesia, speech disorders, praxis, gnosis and pathology of the oral fluid and buccal epithelium, especially with the severity of karyopycnosis and karyorexis, have been established, indicating a direct correlation between the neurodegenerative process pathogenetically associated with Alzheimer’s disease and the processes of systemic inflammation and degeneration of the buccal epithelium.

**Disclosure:**

No significant relationships.

